# Severe neck pain and odynophagia secondary to acute calcific longus colli tendinitis: a case report

**DOI:** 10.1186/s13256-020-02480-z

**Published:** 2020-09-06

**Authors:** Brendan Langford, Jennifer Kleinman Sween, David M. Penn, W. Michael Hooten

**Affiliations:** 1 Department of Anesthesiology and Perioperative Medicine, Mayo Clinic, Rochester, MN USA; 2 Department of Hospital Internal Medicine, Mayo Clinic, Rochester, MN USA; 3 Department of Radiology, Mayo Clinic, Owatonna, MN USA; 4 Department of Anesthesiology and Perioperative Medicine, Division of Pain Medicine, Mayo Clinic, Rochester, MN USA

**Keywords:** Longus colli tendinitis, Neck pain, Calcium crystals, NSAIDs

## Abstract

**Background:**

Acute calcific longus colli tendinitis is a rare, noninfectious inflammatory condition caused by the deposition of calcium crystals. The condition is self-limiting, yet commonly misdiagnosed. Here we report a case of a patient with severe neck pain and odynophagia initially misdiagnosed as a retropharyngeal abscess before establishing the correct diagnosis of acute calcific longus colli tendinitis.

**Case presentation:**

A 60-year-old Caucasian man presented to an outside emergency department with a 5-day history of neck pain and odynophagia. The neck pain was severe and aggravated by movement. Laboratory evaluation revealed leukocytosis and elevated C-reactive protein. Computed tomography of his neck soft tissues was initially interpreted as a retropharyngeal abscess. Antibiotic therapy with piperacillin/tazobactam was initiated, and the patient was transferred to our tertiary care center for further evaluation and treatment. On physical examination, the patient’s neck range of motion was significantly diminished, and bilateral neck tenderness was present. An otolaryngologist performed an examination with laryngoscopy, the result of which was unremarkable. A radiologist at our facility interpreted his outside magnetic resonance imaging as showing “calcification in the prevertebral muscles at C1-C2, inflammation with edema of the prevertebral muscles, and retropharyngeal space edema/effusion,” consistent with acute calcific longus colli tendinitis. His antibiotics were discontinued, and he was started on intravenous ketorolac. He had significant improvement in his neck range of motion, and his pain diminished greatly. He was discharged on a 10-day course of diclofenac (50 mg three times daily). At 1-week follow-up, the patient was doing well; he had returned to work, and his pain was well controlled.

**Conclusions:**

This case report details the presentation, characteristic radiographic findings, and management of a patient with an extremely rare condition of neck pain and odynophagia that could be treated with nonsteroidal anti-inflammatory drugs.

## Background

Acute calcific longus colli tendinitis is a noninfectious inflammatory process secondary to the “deposition of amorphous calcium hydroxyapatite crystals in the tendons of the longus colli muscle,” most commonly anterior to the C1–C2 vertebral level [1]. Symptoms commonly include neck pain, limited cervical range of motion with neck stiffness, and odynophagia. The condition is extremely rare, with an estimated incidence of 0.50 cases per 100,000 person-years [2]. Due to its rarity, acute calcific longus colli tendinitis is often misdiagnosed as a retropharyngeal abscess, herniation, neck tumor, or associated with trauma [3]. Here, we report a case of severe neck pain and odynophagia initially misdiagnosed as a retropharyngeal abscess at an outside hospital before the correct diagnosis of acute calcific longus colli tendinitis was established.

## Case presentation

A 60-year-old Caucasian man with a past medical history significant for hypertension, obstructive sleep apnea, tobacco use, solitary pulmonary nodule, and hyperlipidemia presented to the emergency department as a transfer from an outside facility for further evaluation of neck pain concerning for retropharyngeal abscess based on imaging and clinical presentation. He presented with a 5-day history of sudden-onset neck and postauricular pain that occurred at rest. The pain was constant and was rated 9 on a 10-point numeric pain scale (0 = no pain; 10 = most severe possible pain). His pain intensity was amplified by neck movement. He also endorsed a 4-day history of sore throat and odynophagia associated with a change in voice quality. Two days prior to his presentation, he was evaluated at his local primary care clinic and was diagnosed with “right neck strain and spasm with probable cervical radiculopathy” and suspected bacterial pharyngitis. He was treated with rest, ice, ibuprofen, and cyclobenzaprine. For the suspected bacterial pharyngitis, he was prescribed a 10-day course of amoxicillin-clavulanate.

The following day, he presented to an outside emergency department due to progression of symptoms. Laboratory testing revealed leukocytosis of 13,000 cells/mm^3^ and elevated C-reactive protein of 44.3 mg/L. The results of his basic metabolic panel (including sodium, potassium, and creatinine levels) and lactate measurement were normal. Influenza, respiratory syncytial virus, methicillin-resistant *Staphylococcus aureus* culture, and rapid group A *Streptococcus* test results were all negative. Blood cultures were drawn. Computed tomography (CT) of the head revealed no acute intracranial findings. CT of the neck soft tissue was initially interpreted as showing a 1.1 × 5.6–cm retropharyngeal abscess with enlarged palatine tonsils, and subsequent magnetic resonance imaging (MRI) initially confirmed a 1 × 7.1 × 3.2–cm prevertebral abscess. He was started on piperacillin/tazobactam and was administered 6 mg of morphine intravenously, followed 2 hours later by 100 μg of fentanyl intravenously. This reduced his pain from 9/10 to a 5/10. The patient was transferred to our tertiary referral center for formal otolaryngology evaluation and possible surgical intervention.

Following transfer to our facility’s emergency department, laryngoscopy was performed by an otolaryngologist, and the findings were unremarkable. The otolaryngologist reviewed the imaging and felt that the retropharyngeal fluid collection was unlikely to be infectious, owing to the lack of rim enhancement. The diagnostic images were also reviewed by members of the spine surgery service, who felt the findings were not consistent with an epidural abscess or infection involving the spine. However, given the lack of diagnostic clarity, intravenous antibiotics were continued, and the patient was admitted to the general medical service.

On admission, the patient’s physical examination was notable for significantly diminished range of motion of his neck in all directions, and there was tenderness to palpation of his neck laterally. Oropharyngeal examination was difficult secondary to body habitus (Mallampati class IV); however, no abnormalities were detected.

**Fig. 1:**
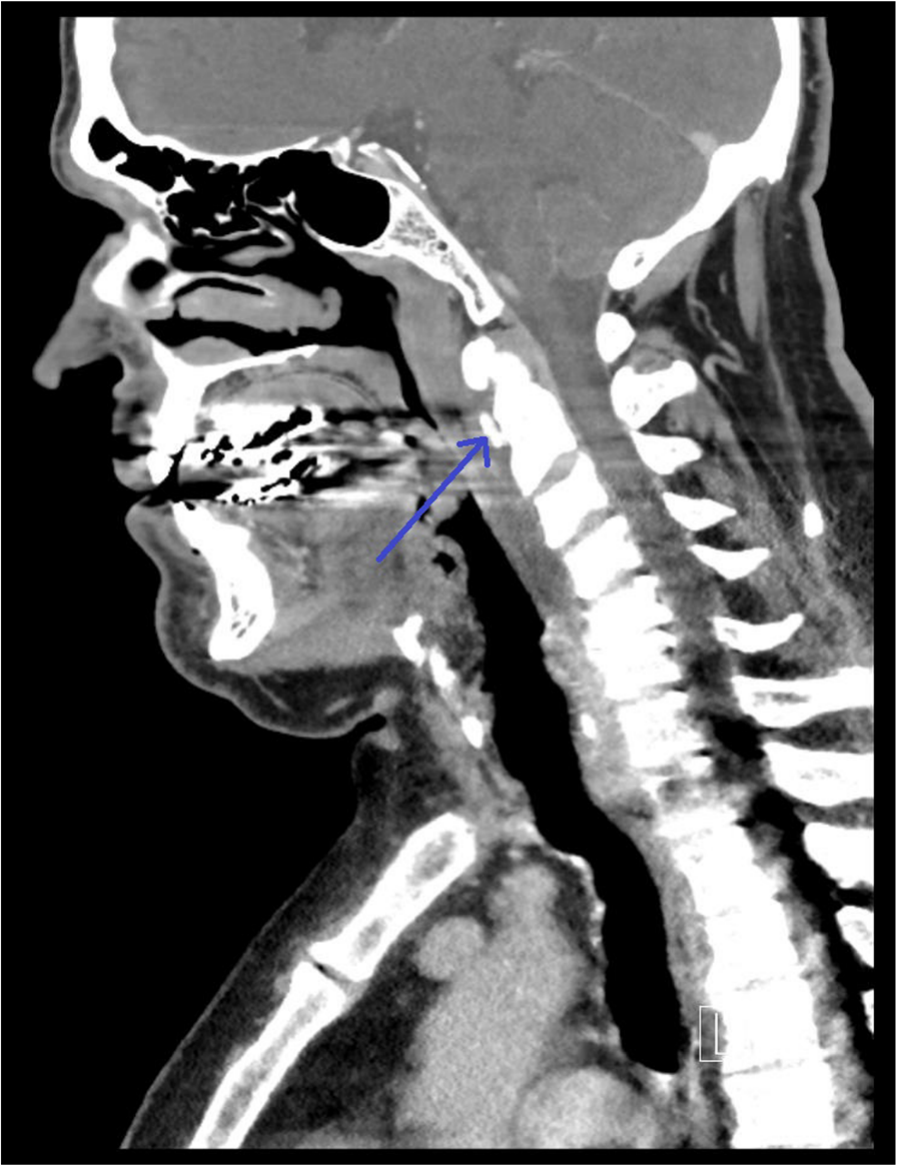
CT of soft tissues of the neck shows a hyperdensity from calcium deposition in the longus colli muscle at the level of C2 (blue arrow)

**Fig. 2:**
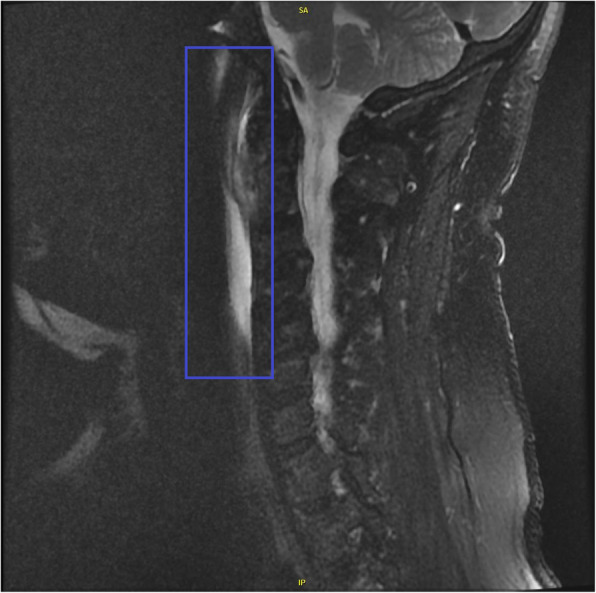
MRI T2 Fat Saturated sequence of cervical spine shows high-signal intensity in the soft tissues representing inflammatory edema of the prevertebral muscles and elongated retropharyngeal effusion (blue rectangle)

His outside images (Figs. 1 and 2) were reviewed by the radiology team at our facility. They stated that CT of the patient’s neck soft tissue exhibited a “retropharyngeal effusion, less likely abscess given [the] lack of [a] thick wall or rim enhancement.” The patient’s MRI scan was reviewed as exhibiting “acute calcific longus colli tendinitis with 1 cm calcification in the prevertebral muscles at C1-C2, inflammation with edema of the prevertebral muscles, and retropharyngeal space edema/effusion.”

Following diagnosis of acute calcific longus colli tendinitis, the medical team discontinued antibiotics and dexamethasone and started the patient on intravenous ketorolac while he remained an inpatient. After starting the ketorolac, he had significant improvement in neck range of motion in all directions, and his pain diminished greatly. He was discharged on a 10-day course of oral diclofenac 50 mg three times daily. His blood culture results remained negative after 5 days of inoculation.

At one-week follow-up, he was able to return to work, and minimal neck stiffness was reported. His pain was adequately controlled with the oral diclofenac.

## Discussion

Acute calcific longus colli tendinitis is a rare, noninfectious inflammatory condition that involves deposition of calcium crystals in the longus colli [1]. The mechanism of calcium deposition is largely unknown; however, it may be secondary to repetitive trauma, recent injury, tissue necrosis, or ischemia [4]. Patients with acute calcific longus colli tendinitis typically have severe neck pain with limited neck range of motion that is accompanied by odynophagia and headache. In a recent case series published in 2017, the majority (62.5%) of patients were males in their fifth decade of life [5]. However, in a separate case analysis of ten patients, only three were male [6]. The age range was 26–68 years, with a mean age of 46.6 years [6].

CT findings of calcium deposition in the longus colli muscle with retropharyngeal edema are sufficient to diagnose acute calcific longus colli tendinitis [7]. MRI examination can also make the diagnosis with findings of calcium deposition, edema, and nonenhancing retropharyngeal effusion [7]. The diagnosis is usually made by CT of the neck soft tissue, which is ordered with the clinical presumption of pharyngitis to rule out abscess [8]. In the setting of retropharyngeal abscess, the fluid will rim enhance and be rounded in shape. In noninfectious retropharyngeal effusion, the fluid will not enhance and will be elongated. In the setting of infectious spondylodiscitis/osteomyelitis, the disc will enhance, the vertebral body endplates will have erosions, and there may be an epidural abscess. Early diagnosis of acute calcific longus colli tendinitis and distinction from these other pathologies may prevent unnecessary antibiotic administration and surgery.

This case report presents the radiographic findings associated with the condition in order for physicians to be more comfortable in establishing its diagnosis. Nonsteroidal anti-inflammatory drugs (NSAIDs) are thought to be effective in treating acute calcific longus colli tendinitis; however, there are no formal treatment guidelines [9]. Acute calcific longus colli tendinitis is a self-limiting condition that will resolve in 1–2 weeks and does not require follow-up imaging after initial diagnosis [6].

## Conclusion

This case report details the presentation, radiographic findings, and management of a patient with an extremely rare condition of neck pain and odynophagia. Despite the lack of formal treatment guidelines for this condition, NSAIDs are known to help decrease the inflammatory process seen in acute calcific longus colli tendinitis.

## Data Availability

Not applicable.

## Abbreviations

CT: Computed tomography
MRI: Magnetic resonance imaging
NSAID: Nonsteroidal anti-inflammatory drug
